# Identification of the Tumor Immune Microenvironment and Therapeutic Biomarkers by a Novel Molecular Subtype Based on Aging-Related Genes in Hepatocellular Carcinoma

**DOI:** 10.3389/fsurg.2022.836080

**Published:** 2022-03-22

**Authors:** Dong Cai, Zhibo Zhao, Jiejun Hu, Xin Dai, Guochao Zhong, Jianping Gong, Feng Qi

**Affiliations:** Department of Hepatobiliary Surgery, The Second Affiliated Hospital of Chongqing Medical University, Chongqing, China

**Keywords:** hepatocellular carcinoma (HCC), immune cells, immune checkpoints, tumor immune microenvironment (TIME), aging, therapeutic biomarkers

## Abstract

**Background:**

Hepatocellular carcinoma (HCC) is one of the most prevalent malignant tumors with poor prognosis. Increasing evidence has revealed that immune cells and checkpoints in the tumor microenvironment (TME) and aging are associated with the prognosis of HCC. However, the association between aging and the tumor immune microenvironment (TIME) in HCC is still unclear.

**Methods:**

RNA expression profiles and clinical data concerning HCC were downloaded from The Cancer Genome Atlas (TCGA) and Gene Expression Omnibus (GEO) databases. Based on differentially expressed aging-related genes (DEAGs), unsupervised clustering was used to identify a novel molecular subtype in HCC. The features of immune cell infiltration and checkpoints were further explored through CIBERSORTx. Enrichment analysis and both univariate and multivariate Cox analyses were conducted to construct a 3-gene model for predicting prognosis and chemosensitivity. Finally, the mRNA and protein expression levels of the 3 genes were verified in HCC and other cancers through database searches and experiments.

**Results:**

Eleven differentially expressed AGs (GHR, APOC3, FOXM1, PON1, TOP2A, FEN1, HELLS, BUB1B, PPARGC1A, PRKDC, and H2AFX) correlated with the prognosis of HCC were used to divide HCC into two subtypes in which the prognosis was different. In cluster 2, which had a poorer prognosis, the infiltration of naive B cells and monocytes was lower in the TCGA and GEO cohorts, while the infiltration of M0 macrophages was higher. In addition, the TCGA cohort indicated that the microenvironment of cluster 2 had more immunosuppression through immune checkpoints. Enrichment analysis suggested that the MYC and E2F targets were positively associated with cluster 2 in the TCGA and GEO cohorts. Additionally, 3 genes (HMGCS2, SLC22A1, and G6PD) were screened to construct the prognostic model through univariate/multivariate Cox analysis. Then, the model was validated through the TCGA validation set and GEO dataset (GSE54236). Cox analysis indicated that the risk score was an independent prognostic factor and that patients in the high-risk group were sensitive to multiple targeted drugs (sorafenib, gemcitabine, rapamycin, etc.). Finally, significantly differential expression of the 3 genes was detected across cancers.

**Conclusion:**

We systematically described the immune differences in the TME between the molecular subtypes based on AGs and constructed a novel three-gene signature to predict prognosis and chemosensitivity in patients with HCC.

## Introduction

The morbidity of hepatocellular carcinoma (HCC) ranks sixth among all cancers, and the mortality of HCC ranks second (1). Currently, HCC therapies include hepatic resection, liver transplantation, transarterial chemoembolization, ablation, and targeted therapies (2). Unfortunately, these therapies are not always satisfactory due to the high malignancy or heterogeneity of HCC in some patients (3, 4). Thus, there is an urgent need to screen new biomarkers of HCC to identify patients with high risk, poor prognosis, and high mortality.

Aging is an essentially universal characteristic of living organisms and is associated with the development of tumors, including HCC (5–8). Cellular senescence plays a dual role in the development of tumors, and its mechanisms in tumors are extremely complex. The senescence-associated secretory phenotype (SASP) contributes to this phenomenon, which means that senescent cells can secrete a variety of cytokines, chemokines, growth factors, and proteases to promote or inhibit tumor growth by enforcing arrest or regulating immune clearance (9). For example, oncogene activation in mammalian cells results in proliferative stress and senescence induction that limits tumor growth, which is also called oncogene-induced senescence (OIS) (10). Braig et al. found that the overexpression of RAS without additional oncogenes and tumor suppressors works as a barrier to block tumor growth *in vivo* (11). Interestingly, some senescent cells can secrete factors that create an immunosuppressive environment to promote tumor growth, and Toso et al. found that SASP factors produced by senescent prostate cells were immune-suppressive cytokines, such as CXCL2 and GMCSF, which are typically activated by signal transducer and activator of transcription 3 (STAT3) (12). In addition, the tumor microenvironment (TME) is one of the crucial factors of tumor growth, which establishes a niche for cancer cells, multiple stromal cells (endothelial cells, immune cells, etc.), and extracellular components (extracellular matrix, cytokines, growth factors, etc.) (13). Indeed, some studies have shown that the SASP of senescent cells contributes to the TME by cytokines (IL-6, CCL2, CCL5, etc.) (14–16). Kang et al. found that senescent cells stimulated the strong response of CD4^+^ T cells in the liver of mice with activated Ras expression (17). Aging-related genes (AGs) can regulate cellular senescence and play a key role in tumor malignancy (7, 18). Due to the dual role of cellular senescence and tumor heterogeneity, it is necessary to explore new biomarkers and molecular mechanisms of aging to better assess prognosis in different HCC patients. Moreover, there is still a lack of effective prognostic models based on AGs to systematically explore the intrinsic molecular differences and TME in HCC.

Thus, the present study divided HCC patients into different subtypes based on survival-associated AGs. Subsequently, we systematically described the differences in clinical characteristics, immune cells in the TME, immune checkpoints, gene expression, and their potential biological functions between different subtypes. Then, we identified 3 survival-associated genes (HMGCS2, SLC22A1, and G6PD) between subtypes to construct a new prognostic model in HCC. Finally, we explored the correlation between the 3 genes and immune cells in the TME and their expression in HCC and multiple cancers. Our study systematically described the differences between subtypes based on AGs and revealed underlying implications as biomarkers for predicting the clinical prognosis of HCC patients.

## Materials and Methods

### Data Downloading and Preprocessing

RNA sequencing data [count and fragments per kilobase million (FPKM) data] and clinical follow-up information for liver hepatocellular carcinoma (LIHC) were obtained from The Cancer Genome Atlas (TCGA) database. The GSE14520 and GSE54236 chip datasets containing survival time information were downloaded from the Gene Expression Omnibus (GEO) database. AGs were acquired from the human aging genome resource dataset [http://genomics.senescence.info/genes/ (19)].

The TCGA-LIHC RNA sequencing and GEO data were preprocessed as follows: (1) the samples with no clinical features [including sex, age, and tumor node metastasis (TNM) staging] information were removed; (2) the samples with no survival time information were removed; and (3) the samples with no status information were removed. We ultimately obtained 341 TCGA-LIHC patients and 219 (GSE14520) and 81 (GSE54236) HCC patients.

Then, we used R to randomly divide TCGA-LIHC patients (*n* = 340) into the training set (*n* = 170), and the validation set (*n* = 170) according to the training set-to-validation set ratio of 1:1. Then, the FPKM value from TCGA was converted into the TPM value through R for subsequent analysis.

### Identification of Differentially Expressed Genes

The differentially expressed genes (DEGs) between tumor and non-tumor samples were calculated by using the limma package in R and filtered according to the threshold | log_2_ [fold change (FC)] | ≥ 1 and adjusted *p* < 0.05. Then, an online Venn diagram website (http://bioinformatics.psb.ugent.be/webtools/Venn/) was used to determine the intersecting genes.

### Identification of Molecular Subtypes

The expression levels of 34 differentially expressed AGs and clinical information were extracted from the TCGA training set and GEO (GSE14520) dataset. Univariate Cox analysis was used to identify the survival-associated AGs in the TCGA training set and the GEO (GSE14520) dataset according to the standard of *p* < 0.05. Then, 11 genes were obtained by the intersection of the TCGA training set and GEO (GSE14520) dataset. The ConsensusClusterPlus package of R was used to cluster the samples consistently according to the above 11 genes (parameters: reps = 50, pItem = 0.8, pFeature = 1, distance = Pearson).

### Estimating the Compositions of Immune Cells

CIBERSORT employs a deconvolution algorithm based on the principle of linear support vector regression used to describe the infiltration of immune cells in the sample (20). LM22 is composed of 547 genes that accurately distinguish 22 human hematopoietic cell phenotypes, such as seven T cell types, naive and memory B cells, plasma cells, natural killer (NK) cells, and myeloid subsets (20). CIBERSORT is a popular algorithm that was extensively utilized in medical studies (21–24). CIBERSORTx was used to estimate the compositions of 22 human immune cell types in the TCGA training set and GEO (GSE14520) dataset. For each sample, the sum of all estimated immune cell type scores was equal to 1.

### Enrichment Analyses and Construction of the Protein–Protein Interactions Network

The results of the gene set enrichment analysis (GSEA) were obtained through GSEA software (V4.1.0). The results of Gene Ontology (GO) and Kyoto Encyclopedia of Genes and Genomes (KEGG) functional enrichment analyses were acquired through the DAVID database (V6.8).

We employed the search tool for recurring instances of neighbouring genes (STRING) database (V11.5) with validated and conjectural protein–protein interactions (PPIs) to obtain the corresponding PPI network. Subsequently, the MCODE clustering algorithm in Cytoscape (V3.7) was used for subnet screening (K-core ≥ 7, node score cutoff = 0.2, degree cutoff = 2, and max depth = 100) (25–27).

### Construction and Verification of the Prognostic Risk Model

Univariate and multivariate Cox proportional hazard regression analyses were carried out using SPSS (V20) to identify the final signatures for the risk model, and *p* < 0.05 was selected as the threshold value. A multigene prognostic risk score was established based on a combination of regression coefficients from the multivariate Cox regression model (β) multiplied by their mRNA expression levels. Risk score = β x expression of G6PD + β x expression of HMGCS2 + β x expression of SLC22A1. The median was used as a cutoff value to divide HCC patients into high- and low-risk groups. The Kaplan–Meier (KM) survival curves and time-dependent receiver operating characteristic (ROC) curve analyses were performed to assess the predictive capacity of the model.

### Gene Expression Validation in HCC and Pancancer

We compared the mRNA or protein expression levels of the two genes in HCC and normal tissues *via* Gene Expression Profiling Interactive Analysis (GEPIA) and the Human Protein Atlas (HPA), respectively. Then, we performed western blotting to further verify the protein expression of 3 genes with anti-G6PD (ab210702), anti-HMGCS2 (ab137043), and anti-SLA22A1 (ab181022) antibodies (Abcam). Finally, we obtained the mRNA expression levels of the 3 genes across cancers through the GEPIA database.

## Results

### Construction of Molecular Subtyping Based on AGs and Comparison of Clinical Features Between Subtypes

**Figure 1:**
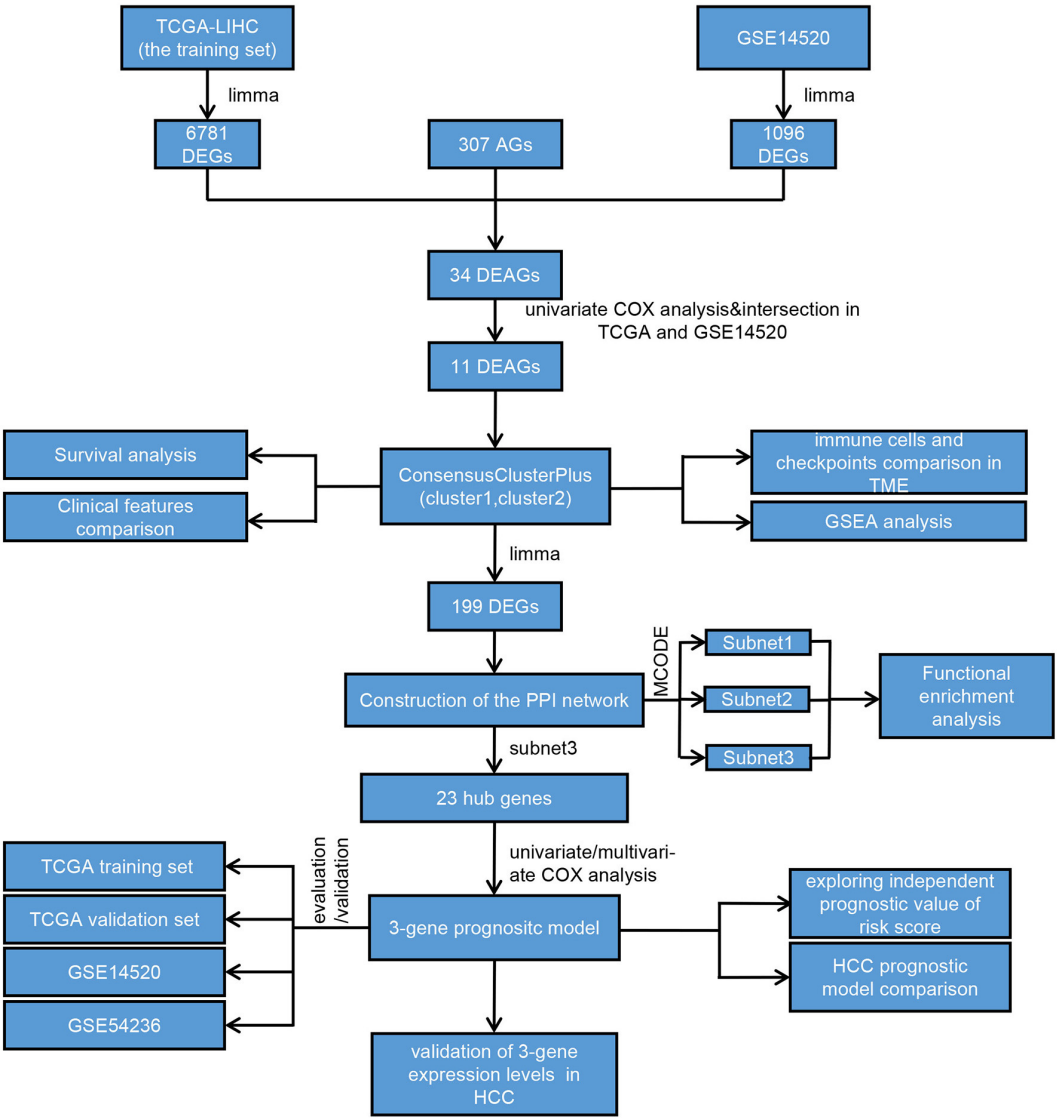
Flowchart of the overall study.

**Figure 2:**
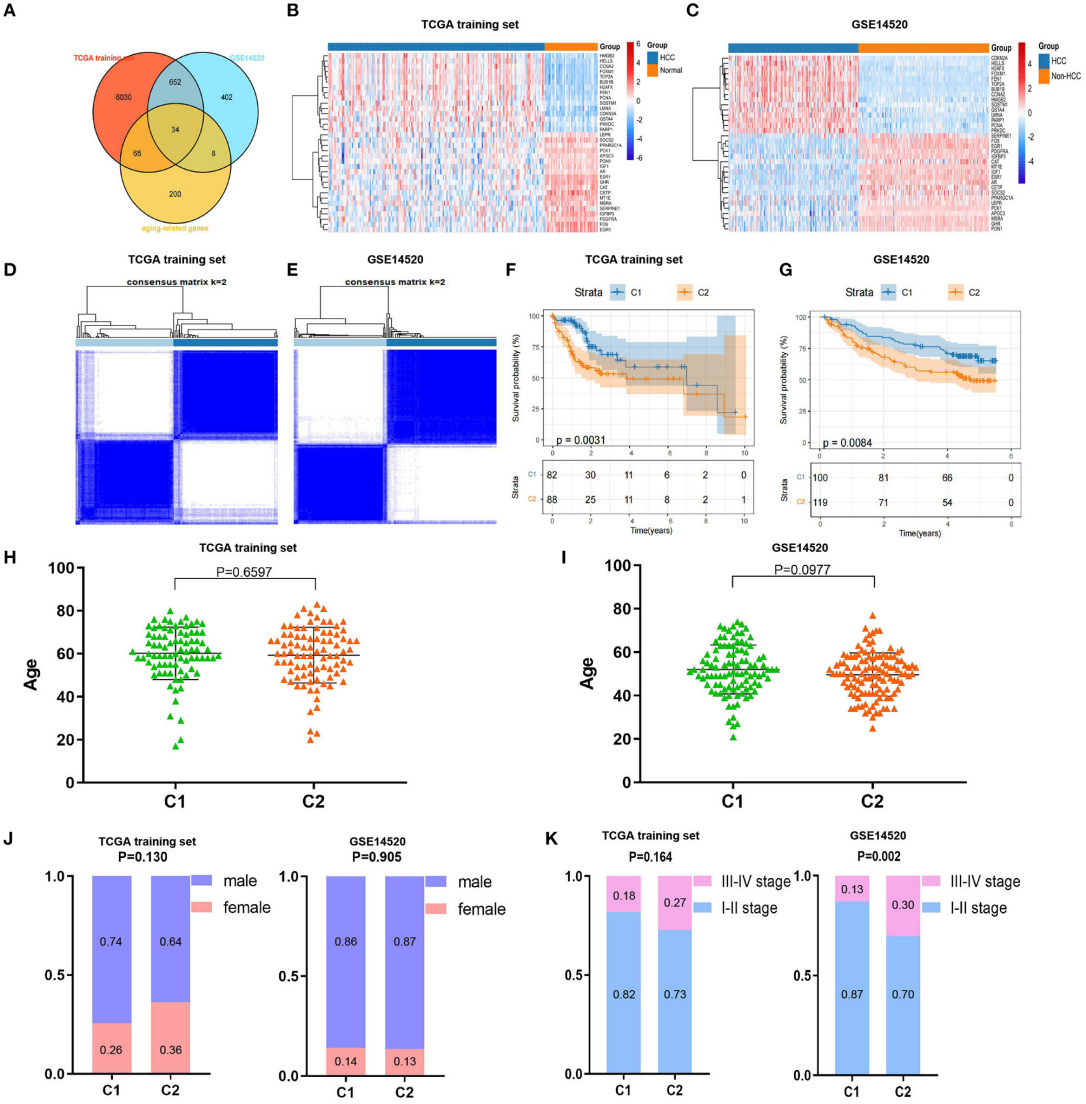
Screening of differentially expressed aging-related genes (DEAGs), consistent clustering analysis, and comparison of molecular subtypes. **(A)** Venn diagram of DEGs from the The Cancer Genome Atlas (TCGA) training set, DEGs from GSE14520 and AGs. **(B,C)** Heatmaps of 34 DEAGs. **(D,E)** Cluster heatmaps of samples with consistent clusters of *k* = 2. **(F,G)** Kaplan–Meier (KM) curve analysis of overall survival between C1 and C2. **(H,I)** Comparison of age between C1 and C2. **(J)** Comparison of sex between C1 and C2. **(K)** Comparison of TNM stage between C1 and C2.

We drew a methodology flow chart to make the research easier for readers to understand (Figure 1); the clinical information for the all datasets is presented in Table 1. First, we identified 6,787 and 1,096 DEGs based on TCGA (training set) and GEO (GSE14520) databases, respectively. Then, we identified 34 differentially expressed aging-related genes (DEAGs) through the intersection of 6,781 DEGs from TCGA (the training set), 1096 DEGs (GSE14520) from GEO, and 307 AGs (Figures 2A–C). By performing univariate Cox analysis of the TCGA (the training set) and GEO (GSE14520) datasets, we ultimately identified 11 DEAGs (GHR, FOXM1, PON1, TOP2A, FEN1, HELLS, BUB1B, PPARGC1A, PRKDC, and H2AFX) correlated with the prognosis of patients with HCC (*P* < 0.05) for subsequent determination of the molecular subtypes of HCC (Supplementary Table 1).

**Table 1:** Clinical information of HCC patients.

**Clinical features**	**TCGA**		**GSE14520** **(*n* = 219)**	**GSE54236** **(*n* = 81)**
	**The training set** **(*n* = 170)**	**The validation set** **(*n* = 170)**		
**Gender**				
Male	117 (0.69)	116 (0.68)	189 (0.86)	64 (0.79)
Female	53 (0.31)	54 (0.32)	30 (0.14)	17 (0.21)
**Age**				
≥65	67 (0.39)	65 (0.38)	24 (0.11)	–
<65	103 (0.61)	105 (0.62)	195 (0.89)	–
**TNM stage**				
I	87 (0.51)	82 (0.48)	93 (0.43)	–
II	44 (0.26)	40 (0.24)	77 (0.35)	–
III	37 (0.22)	46 (0.27)	49 (0.22)	–
IV	2 (0.01)	2 (0.01)	0 (0)	–
**Status**				
Alive	112 (0.66)	113 (0.66)	135 (0.62)	0 (0)
Dead	58 (0.34)	57 (0.34)	84 (0.38)	81 (1)

Consistent cluster analysis was performed and indicated that the samples of the TCGA training set and GEO (GSE14520) dataset could be clustered at *k* = 2 based on the expression profiles of the above 11 DEAGs (Figures 2D,E, Supplementary Figure 1). KM curves indicated that patients in C2 (cluster 1) had a worse prognosis than patients in C1 (cluster 2) for the TCGA training set (*p* = 0.0031, Figure 2F). Moreover, patients in C2 (cluster 2) had a worse prognosis than patients in C1 (cluster 1) for the GEO dataset (*p* = 0.0084, Figure 2G).

Furthermore, we compared the distributions of different clinical features between the C1 and C2 subtypes and found that there was no significant difference in age or sex between the C1 and C2 subtypes for the TCGA training and GEO datasets (Figures 2H–J). Notably, we found that there were significantly more patients with stage III–IV disease in the C2 subtype (*p* = 0.002) for the GEO dataset and a similar trend in the TCGA training set, although the difference was not statistically significant (Figure 2K). These results showed that patients in the C2 subtype of the TCGA training set and GEO dataset had worse prognoses.

### Comparison of Immune Cell Infiltration and Immune Checkpoints Between Subtypes in the TME

**Figure 3:**
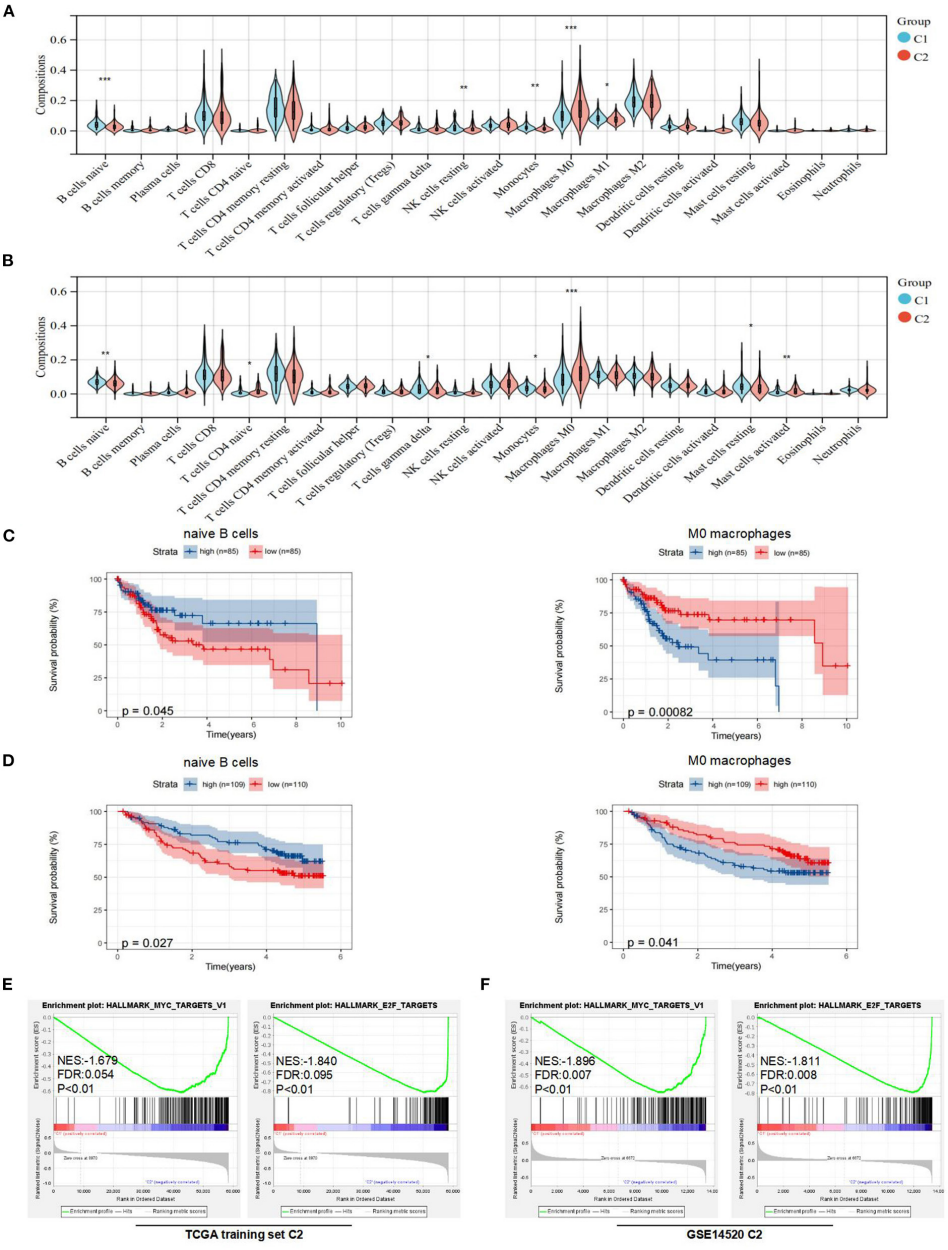
Analysis of immune features and gene set enrichment analysis (GSEA) between subtypes. **(A)** Comparison of immune cells in the tumor microenvironment (TME) between C1 and C2 in the TCGA training set. **(B)** Comparison of immune cells in the TME between C1 and C2 in the Gene Expression Omnibus (GEO) dataset. **(C)** KM curve analysis of overall survival between high and low compositions of immune cells (naive B cells and M0 macrophages) in the TCGA training set. **(D)** KM curve analysis of overall survival between high and low compositions of immune cells (naive B cells and M0 macrophages) in the GSE14520. **(E)** GSEA analysis in the TCGA training set. **(F)** GSEA analysis in the GSE14520. *p < 0.05, **p < 0.01, and ***p < 0.001.

To further explain the differences in prognoses between subtypes, we explored the compositions of immune cells and the expression of immune checkpoints in the TME between subtypes. First, we used CIBERSORTx to obtain the compositions of 22 immune cell types in the TME (Supplementary Figures 2A,B). Then, we found that the compositions of naive B cells, monocytes, resting NK cells, M0 macrophages, and M1 macrophages were significantly different between the two subtypes in the TCGA training set (Figure 3A). Similarly, the compositions of naive B cells, monocytes, naive CD4^+^ T cells, gamma delta T cells, M0 macrophages, and resting and activated mast cells were significantly different between the two subtypes in the GEO dataset (Figure 3B).

To determine underlying molecular hallmarks leading to poor prognosis in the TCGA training set and GEO data set. Firstly, the median was used as the cutoff value and KM analysis of 3 immune cell types (naive B cells, monocytes, and M0 macrophages) indicated that naive B cells and M0 macrophages played the more important roles in the prognosis of HCC (Figures 3C,D, Supplementary Figures 2C,D). Furthermore, the expression of immune checkpoints was compared between the two subtypes. The results indicated that the expression levels of PD-1, CTLA-4, LAG-3, and TIM-3 were all markedly higher (*p* < 0.05) in the C2 subtype in the TCGA training set. For the GEO dataset, the expression levels of PD-1 were higher (*p* < 0.05) in the C1 subtype, while there was no difference in the expression of CTLA-4 and LAG-3 between the two subtypes (Supplementary Figure 2E). Unfortunately, no expression of TIGIT or TIM-3 was detected in GSE14520.

In addition, the GSEA indicated that E2F and MYC targets were positively associated with poor prognostic subtypes in the TCGA training set. Similarly, we obtained the same results in the GSE14520 cohort (Figures 3E,F). More detailed GSEA results can be found in Supplementary Figure 3. Taken together, these results suggested that subtypes with poor prognoses in the TCGA training set and GEO dataset had similar malignant hallmarks.

### Identification of DEGs and Screening and Functional Analysis of Hub Genes Between Subtypes

**Figure 4:**
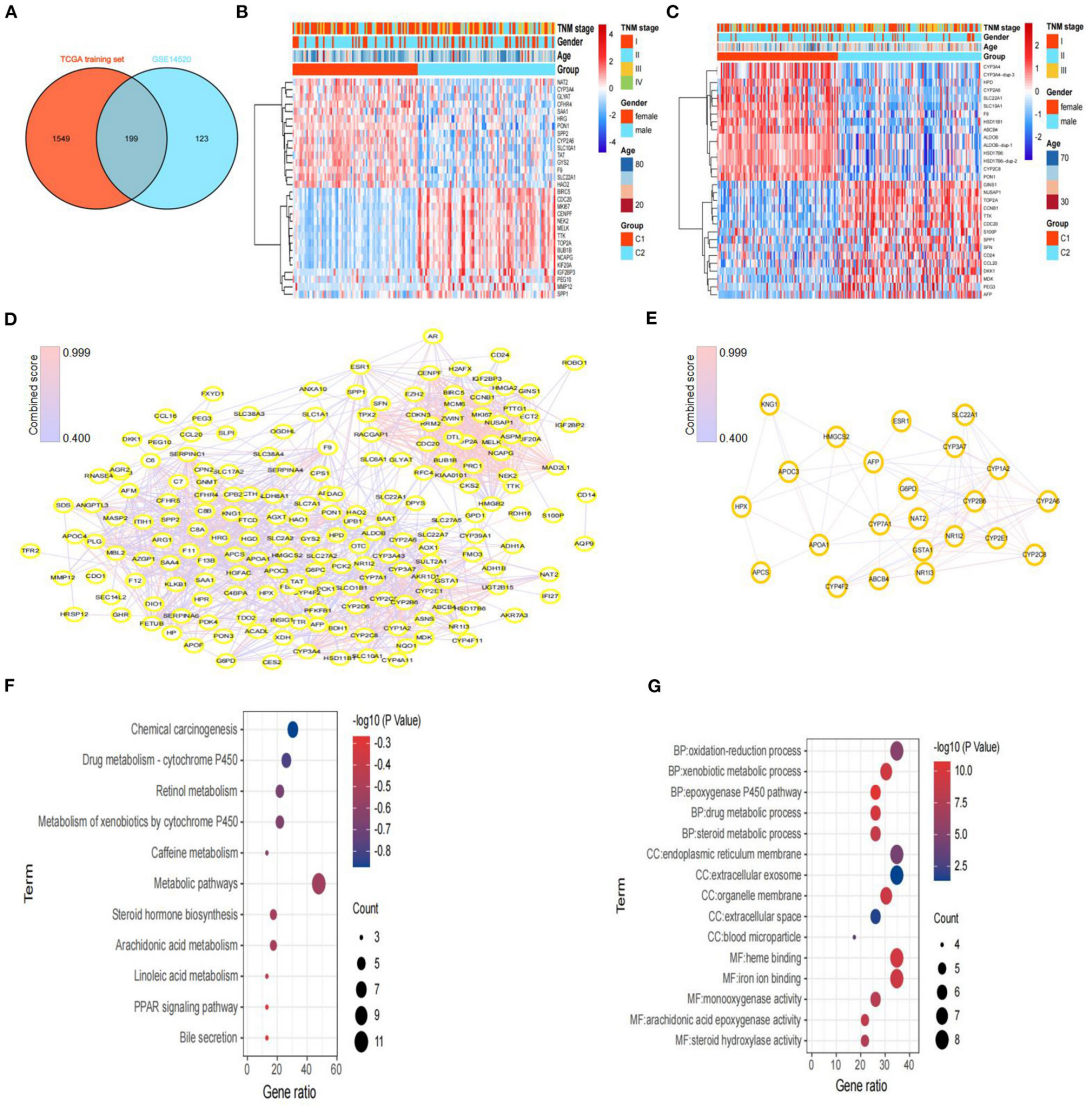
Screening of DEGs and hub genes between subtypes and functional enrichment analysis. **(A)** Venn diagram of DEGs between subtypes from the TCGA training set and DEGs from GSE14520. **(B)** Heatmaps of the top 30 DEGs between C1 and C2 in the TCGA training set. **(C)** Heatmaps of the top 30 DEGs between C1 and C2 in GSE14520. **(D)** Protein–protein interaction (PPI) network based on the 199 DEGs, constructed *via* STRING. **(E)** The PPI network of subnet 3 (hub genes) through MCODE. **(F)** The results (*p* < 0.05) of KEGG analysis based on hub genes. **(G)** The top 5 results of GO analysis (BP, biological processes; CC, cellular components; and MF, molecular functions) based on hub genes.

To further explore the molecular differences between subtypes, we used the limma package in R to identify the DEGs between the C1 and C2 subtypes of the TCGA training set and GEO dataset. Next, we obtained 199 DEGs for the subsequent enrichment analysis through intersection of DEGs of the TCGA training set and DEGs of the GEO dataset (Figure 4A). Interestingly, we also found similar expression levels of these 199 DEGs in subtypes with poor prognoses, and we showed the top 30 DEGs in the TCGA training set and GEO dataset (Figures 4B,C).

Then, we constructed a PPI network through the STRING database; the network had 198 nodes, 1,562 edges, and an average node degree of 15.8 (Figure 4D). Furthermore, we identified 3 subnets (Figure 4E, Supplementary Figure 4A) of the PPI network through the MCODE clustering algorithm and performed KEGG analysis on the four subnets (Supplementary Figure 4B). Notably, the KEGG pathway analysis results showed that the genes of subnet 3 were significantly enriched in metabolic pathways, bile secretion, retinol metabolism, the PPAR signaling pathway, and chemical carcinogenesis (Figure 4F). Thus, we chose genes in subnet 3 as the hub genes. In addition, the top 5 results of GO analysis (biological processes, cellular components, and molecular functions) are presented in Figure 4G.

### Construction and Verification of the Risk Model

The univariate Cox proportional hazard regression model method was used to evaluate the hub genes, and 7 genes (HMGCS2, KNG1, SLC22A1, NR1I2, APOC3, HPX, and G6PD) were found to be associated with prognosis (Supplementary Figure 5A). Then, a multivariate Cox proportional hazard regression model was performed to further narrow the range of variables. Finally, we identified the three genes (HMGCS2, SLC22A1, and G6PD) associated with prognosis to construct the risk model (Supplementary Figure 5B).

**Figure 5:**
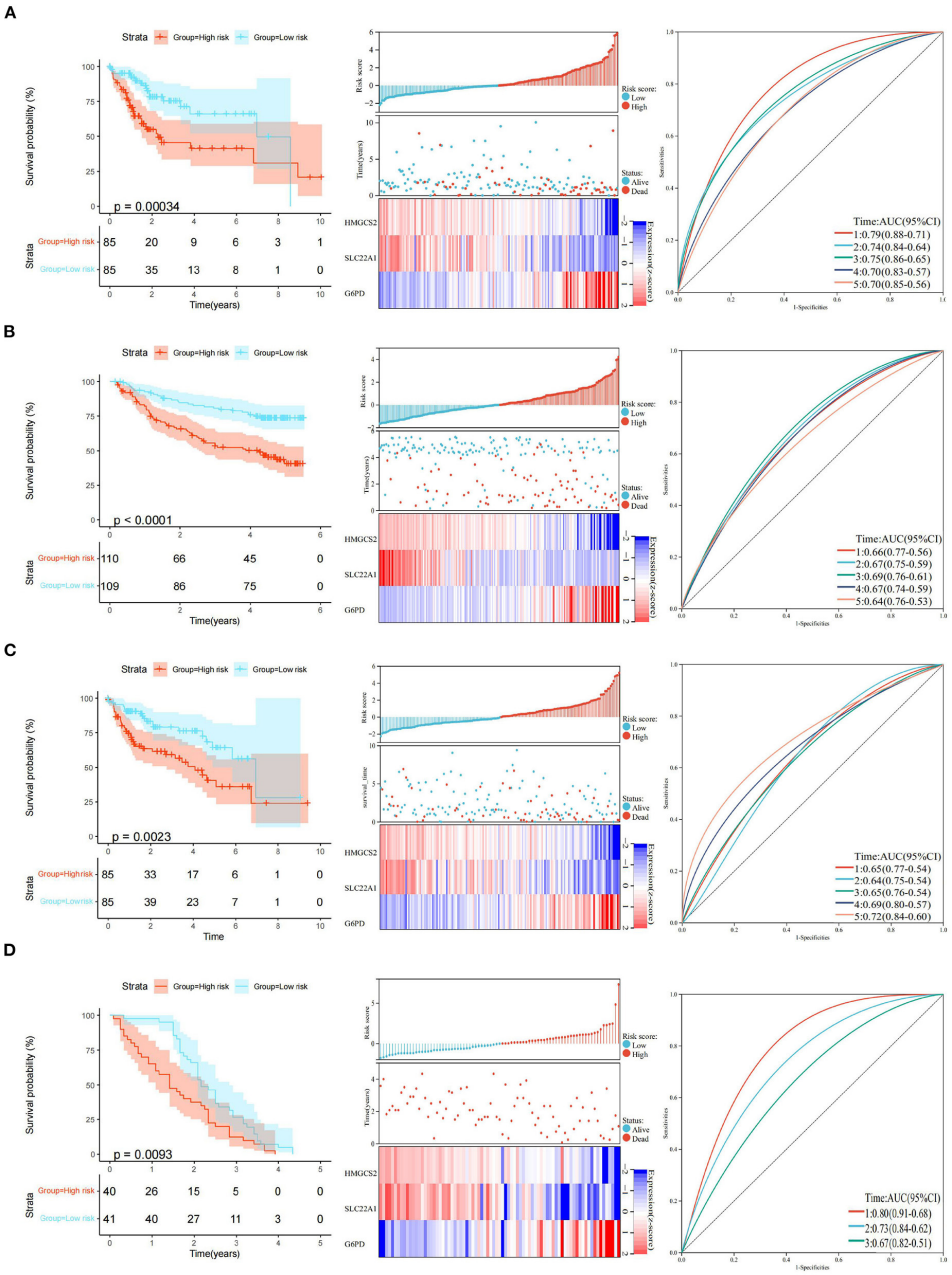
Construction and validation of the 3-gene prognostic model. **(A)** KM curve analysis, distributions of high- and low-risk scores, and time-dependent ROC curve analyses of the TCGA training set. **(B)** KM curve analysis, distributions of high- and low-risk scores, and time-dependent ROC curve analyses in GSE14520. **(C)** KM curve analysis, distributions of high- and low-risk scores, and time-dependent ROC curve analyses of the TCGA validation set. **(D)** KM curve analysis, distributions of high- and low-risk scores, and time-dependent ROC curve analyses in GSE54236.

The final 3-gene signature formula was as follows: Risk score = (−0.426) x HMGCS2 + (−0.314) × SLC22A1 + 0.413 × G6PD. Risk scores were further converted into Z scores. Patients with scores > 0 were divided into the high-risk group, and patients with scores <0 were divided into the low-risk group in the TCGA training set. The KM curves revealed that the patients in the high-risk group had a poorer prognosis (*p* < 0.001) than those in the low-risk group in the TCGA training set. The areas under the time-dependent ROC curve (AUCs) of the TCGA training set at 1, 2, 3, 4, and 5 years were 0.79, 0.74, 0.75, 0.70, and 0.70, respectively (Figure 5A). Similarly, we did the same for the GEO dataset and obtained a similar result (*p* < 0.0001). The areas under the AUC of the GEO dataset at 1, 2, 3, 4 and 5 years were 0.66, 0.67, 0.69, 0.67, and 0.64, respectively (Figure 5B).

The robustness of the model was verified by the internal dataset (the TCGA validation set) and external dataset (GSE54236 dataset). In all the datasets, the same model and coefficients as those used with the TCGA training set were used. The risk score of each patient was calculated according to gene expression, and the distributions of the risk scores were plotted in the TCGA internal validation set. The results showed significant differences (*p* < 0.01) between the high- and low-risk groups in the TCGA validation set. The areas under the AUC of the TCGA validation set at 1, 2, 3, 4, and 5 years were 0.65, 0.64, 0.65, 0.69, and 0.72, respectively (Figure 5C).

Similarly, we divided patients with scores > 0 into the high-risk group and patients with scores <0 into the low-risk group in the GEO external data set. KM curves showed that the patients in the high-risk group had a poorer prognosis (*p* < 0.01) than those in the low-risk group in the GEO external dataset. Interestingly, the areas of AUC at 1, 2, and 3 years in the GEO external dataset were 0.80, 0.73, and 0.67, respectively (Figure 5D). Although we could not obtain an accurate AUC value at 4 and 5 years due to the limitations of the GEO external data set, all of the above results indicate that the model has a certain degree of robustness.

### Independent Prognostic and Therapeutic Value of the Risk Score and Comparison Among HCC Prognostic Models

**Figure 6:**
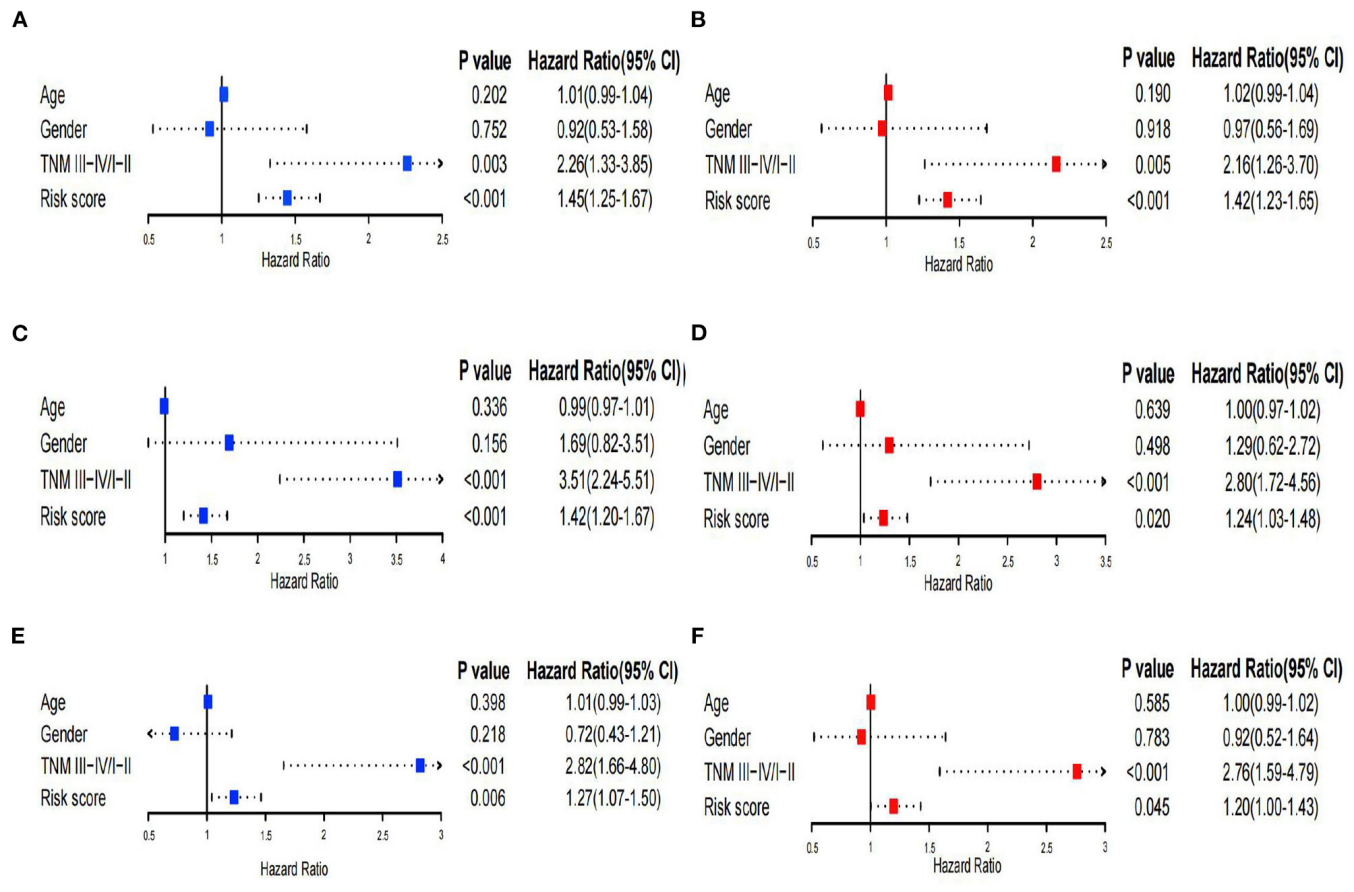
Univariate and multivariate Cox regression analysis. **(A,B)** Univariate and multivariate Cox analyses of the TCGA training set. **(C,D)** Univariate and multivariate Cox analyses of GSE14520. **(E,F)** Univariate and multivariate Cox analyses of the TCGA validation set.

Univariate and multivariate Cox regression analyses were performed to assess independent predictive values for the risk score in HCC patients. In the TCGA training set and GSE14520 dataset, univariate and multivariate Cox regression analyses suggested that the risk score (*p* < 0.001) and TNM staging (*p* = 0.003 and 0.005) had prognostic value, although age and sex were not associated with prognosis (Figures 6A–D). Furthermore, in the TCGA validation set, univariate and multivariate Cox regression analyses also showed that the risk score and TNM staging were independent prognostic values (*p* < 0.05; Figures 6E,F). In addition, we utilized the pRRophetic package to calculate the IC_50_ value between the high- and low-risk groups. In the high-risk group, sorafenib, AKT inhibitor VIII, paclitaxel, gemcitabine, and rapamycin had lower IC_50_ values, while erlotinib had higher IC_50_ values (Supplementary Figures 6A–F). No significant difference in chemosensitivity for axitinib between the two risk groups was observed (Supplementary Figure 6G).

Moreover, we chose several current prognostic models in HCC (28–33) and compared them with our model. Obviously, our 3-gene signature is better than the others. Furthermore, we validated the robustness of our model by using internal and external datasets. We have concluded the results of the comparison in Table 2.

**Table 2:** Comparison among HCC prognostic models.

**Study**	**Our study**	**Zhang et al.** **(28)**	**Chen et al.** **(29)**	**Yang et al.** **(30)**	**Hong et al.** **(31)**	**Liu et al.** **(33)**	**Liang et al.** **(32)**
Signature	3-Gene	14-Gene	7-Gene	4-Gene	11-Gene	6-Gene	10-Gene
**TCGA**							
Training set	*n* = 170	*n* = 312	*n* = 371	*n* = 351	*n* = 171	*n* = 172	*n* = 365
AUC (1, 3, 5 y)	0.79, 0.75, 0.70	0.71, 0.74, 0.64	0.78, 0.76, 0.73	0.80, 0.75, 0.72	0.71, 0.72, 0.81	0.83, 0.85, 0.77	0.80, 0.69, 0.67
Validation set	*n* = 170	**–**	**–**	**–**	*n* = 171	*n* = 171	**–**
AUC (1, 3, 5 y)	0.65, 0.65, 0.72	**–**	**–**	**–**	0.75, 0.73, 0.61	0.71, 0.59, 0.60	**–**
**GEO**							
Validation set	GSE14520 (*n* = 219)	GSE14520 (*n* = 225)	**–**	GSE14520 (*n* = 221)	GSE15654 (*n* = 216)	**–**	**–**
AUC (1, 3, 5 y)	0.66, 0.69, 0.64	0.64, 0.59, 0.65	**–**	0.65, 0.61, 0.63	0.71, 0.58, 0.62	**–**	**–**
	**–**	GSE76427 (*n* = 114)	**–**	**–**	**–**	**–**	**–**
AUC (1, 3, 5 y)	**-**	0.60, 0.64, 0.60					
	GSE54236 (*n* = 81)						
AUC (1, 2, 3 y)	0.80, 0.73, 0.67	0.60, 0.64, 0.60	**–**	**–**	**–**	**–**	**–**
**ICGC**							
Validation set	**–**	**–**	*n* = 260	**–**	*n* = 212	**–**	*n* = 231
AUC (1, 2, 3 y)	**–**	**–**	0.66, 0.72, 0.71	**–**	0.73, 0.71, 0.70	**–**	0.68, 0.69, 0.72

### Correlations of the 3 Genes With Immune Cell Infiltration and Their Expression Across Cancers

**Figure 7:**
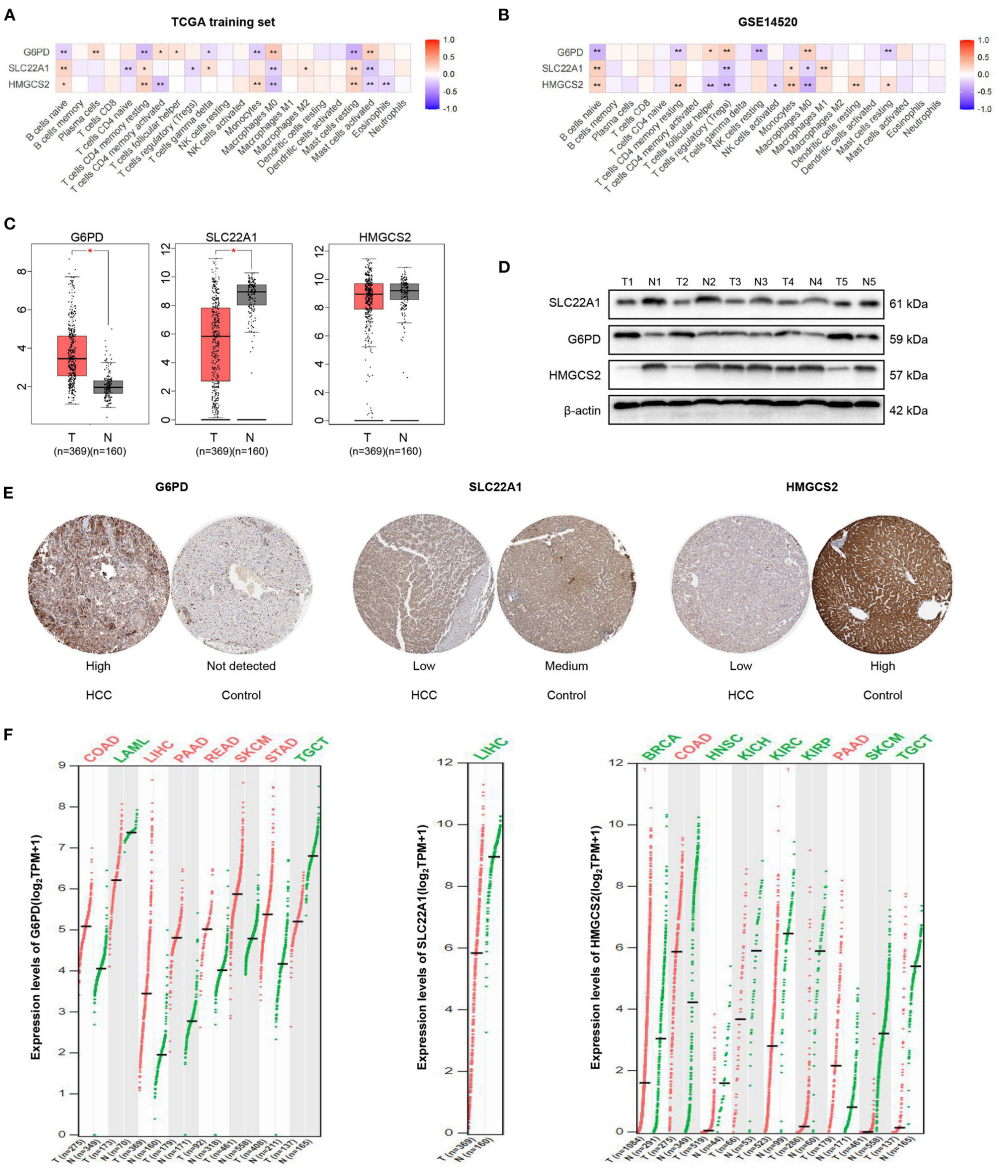
Correlation analysis and expression analysis of the 3 genes. **(A,B)** Correlation of the 3 genes and 22 immune cells. **(C)** mRNA expression levels of the 3 genes in HCC according to GEPIA. **(D)** Protein expression levels of the 3 genes in HCC and tumor-adjacent tissues according to western blotting. **(E)** Immunohistochemical staining of the 3 genes in HPA. **(F)** Comparison of the expression of the 3 genes in different tumors according to GEPIA. The colors represent statistical significance. The red color represents high expression in cancer, and the green color represents low expression in cancer. *p < 0.05; **p < 0.01.

We performed the Spearman's correlation analysis to explore the relationship between the 3 genes (HMGCS2, SLC22A1, and G6PD) and the infiltration of 22 immune cells. The results showed that the expression of the 3 genes was significantly correlated with the compositions of naive B cells and M0 macrophages in the TCGA training set and GEO dataset (Figures 7A,B). This finding may indicate that these 3 genes play important roles in regulating naive B cells and M0 macrophages. Furthermore, our results indicated that G6PD may have the opposite effect on HMGCS2 and SLC22A1, which is consistent with the Cox analysis results.

We then found that the mRNA expression values of G6PD and SLC22A1 were higher or lower in HCC samples than in normal samples by GEPIA, respectively. However, the mRNA expression value of HMGCS2 was not significantly different between HCC and normal samples according to GEPIA, which matches the TCGA normal and GTEx data (Figure 7C). Furthermore, we performed western blotting to validate the protein expression of the 3 genes in 5 HCC and paired tissues. We found that the expression levels of HMGCS2 and SLC22A1 were higher in tumor-adjacent tissues; while the expression levels of G6PD were higher in HCC tissues (Figure 7D). Then, we used immunohistochemical staining in the HPA database to further verify the western blot results (Figure 7E).

In addition, we used GEPIA to show the expression of the 3 genes in 33 cancers compared with corresponding normal samples. Obviously, the expression of G6PD was significantly higher in colon adenocarcinoma (COAD), LIHC, pancreatic adenocarcinoma (PAAD), rectum adenocarcinoma (READ), skin cutaneous melanoma (SKCM), and stomach adenocarcinoma (STAD) but was lower in acute myeloid leukemia (LAML), and testicular germ cell tumors (TGCT). The expression of SLC22A1 was lower in LIHC than in normal samples. In the normal samples, the expression of HMGCS2 was higher in breast invasive carcinoma (BRCA), head and neck squamous cell carcinoma (HNSCC), kidney chromophobe (KICH), kidney renal clear cell carcinoma (KIRC), kidney renal papillary cell carcinoma (KIRP), SKCM and TGCTs (Figure 7F). More detailed expression of the 3 genes in 33 cancers can be found in Supplementary Figure 7 and Supplementary Table 2.

## Discussion

Immune cells, which constitute an important part of the TME, have a crucial effect on the progression of tumors (34). Activated B cells can secrete IL-10 and attenuate antitumor immune responses by regulating T cell responses (35). Macrophages in the TME, as a plastic and heterogeneous cell population, are usually divided into M1-like macrophages (proinflammatory and antitumor) and M2-like macrophages (anti-inflammatory and protumor) (36, 37). Moreover, tumors can alter the functional order of normal macrophages in favor of their own growth, and macrophages are more likely to be M2-like polarized in the TME (38). Indeed, we found that there were more naive B cells in the subtype with the better prognosis, which may contribute to shaping the antitumor TME. In addition, we also found that more M0 macrophages infiltrated the TME, which is beneficial for shaping the immunosuppressive microenvironment. Remarkably, differences in the compositions of naive B cells and M0 macrophages between subtypes were found in both TCGA and GEO databases, which indicated that these cells, but not other cells (CD8^+^ T cells, NK cells, etc.), may play dominant roles in the HCC subtype based on AGs.

Recently, immune checkpoint blockers have become a popular research topic in tumor-targeted therapy, and increasing number of antibodies (nivolumab, atezolizumab, etc.) is used in clinical antitumor therapies (39). Nivolumab is the first FDA-approved anti-PD1 inhibitor for HCC, and a phase 3 randomized trial (NCT02576509) indicated that, as a first-line treatment for advanced HCC, nivolumab treatment was promising compared with sorafenib (40). A clinical trial from Duffy et al. also found that tremelimumab (ancti-CTLA4) combined with ablation is a potential treatment for advanced HCC and leads to the accumulation of CD8^+^ T cells (41). It is with hope that an increased number of immune checkpoints (such as CTLA-4, PD-1, LAG-3, TIM-3, and TIGIT) be documented, although some tumor patients show immunotherapy tolerance (42–44). LAG-3 (CD223), as a checkpoint to prevent overt activation of T cells, is associated with the exhaustion of dysfunctional CD8^+^ T cells (45). TIM-3 (CD366 or HAVCR2) is expressed on highly dysfunctional T cells, and TIM-3-associated drug resistance has been observed in HNSCC and non-small-cell lung cancer (NSCLC) (46, 47). Tan et al. found that the expression of TIM-3 on liver-resident NK (LrNK) cells hampered the function of LrNK cells through PI3K/mTORC1 interference to enhance HCC growth (48). Accumulating evidence has also revealed that TIGIT is highly expressed on T cells, NK cells and M2 macrophages and impairs their antitumor cytotoxicity (49). Consistently, our study also indicated that these immune checkpoints were highly expressed in the TME in the HCC subtype with poor prognosis, which was validated by both TCGA and GEO databases. We hypothesized that the high expression of these immune checkpoints was associated with the differences in the compositions of the above 2 immune cell types (especially naive B cells) in the TME. In summary, our study indicated that the TME in the subtype with poor prognosis was more likely to be immunosuppressive, which may be fundamental to the underlying microenvironment in aging-related tumors.

E2F transcription factors can bind multiple target genes to regulate their expression and are well-known for their function in cell cycle progression and transition into S phase, DNA synthesis, and cellular proliferation (50). Increasing evidence shows that E2F mediates tumor growth and metastasis (51). In our study, the GSEA results suggested that genes involved in the E2F targets may be mainly expressed in the subtype with the worst prognosis. Thus, E2F may be a key point in the molecular mechanisms between the two subtypes based on AGs. Furthermore, the 3 genes may play crucial roles in the HCC subtype based on AGs. In fact, Lu et al. found that, through activation of the STAT3 pathway, elevated expression of G6PD enhanced the migration and invasion of HCC cells (52). HMGCS2 can promote ketone production, which can promote the proliferation of HCC by targeting c-Myc (53). Notably, our GSEA results showed that MYC targets are positively associated with poor prognostic subtypes in HCC, which supported our points that HMGCS2 might play an important role in the development of HCC. Similarly, downregulating SLC22A1 affects the prognosis and response to sorafenib in HCC patients (54, 55).

The most significant advantage of the present work is the construction and validation of a novel three-gene signature model based on AGs in HCC, and we systematically described the immune features in the TME between subtypes. In fact, Chen et al. identified 7 AGs (POLA1, CDK1, SOCS2, HDAC1, MAPT, RAE1, and EEF1E1) and constructed a prognostic model *via* the TCGA and ICGC (international cancer genome consortium) databases (29). However, we not only classified HCC based on AGs, but also explored the immune features of the TME. Moreover, our model had a better AUC value and robustness. Our work is the first to explore aging from the perspective of molecular typing of HCC and to associate it with the TME. In addition, there is a limitation that we need to acknowledge in our study: we lacked prospective clinical data to validate the prognostic significance of the three-gene signature.

Collectively, we classified HCC based on AGs and systematically described the immune features in the TME between subtypes. Then, we constructed and validated a new prognostic model according to the new molecular subtyping. Our work might provide novel insights into the aging and immunity of the TME in HCC.

## Data Availability Statement

All data are from public databases TCGA and GEO. The original contributions presented in the study are included in the article/Supplementary Material, further inquiries can be directed to the corresponding author.

## Author Contributions

DC collected, analyzed and interpreted the data, and drafted the manuscript. ZZ interpreted the data and contributed to the substantial revisions of the manuscript. JH, XD, GZ, and JG helped to perform the statistical analysis and interpreted the data. FQ contributed to the conception and design, analyzed and interpreted the data, supervised the study, and ultimately approved the version of the manuscript for publication. All authors have read and approved the final manuscript.

## Conflict of Interest

The authors declare that the research was conducted in the absence of any commercial or financial relationships that could be construed as a potential conflict ofinterest.

## Publisher's Note

All claims expressed in this article are solely those of the authors and do not necessarily represent those of their affiliated organizations, or those of the publisher, the editors and the reviewers. Any product that may be evaluated in this article, or claim that may be made by its manufacturer, is not guaranteed or endorsed by the publisher.
