# Supplementary material for: Identification of the Tumor Immune Microenvironment and Therapeutic Biomarkers by a Novel Molecular Subtype Based on Aging-Related Genes in Hepatocellular Carcinoma
Source: Front Surg. 2022 Mar 22;9:836080. doi: 10.3389/fsurg.2022.836080 (PMC8980463; doi:10.3389/fsurg.2022.836080)
Supplement: Supplementary file 1 [file Data_Sheet_1.docx]

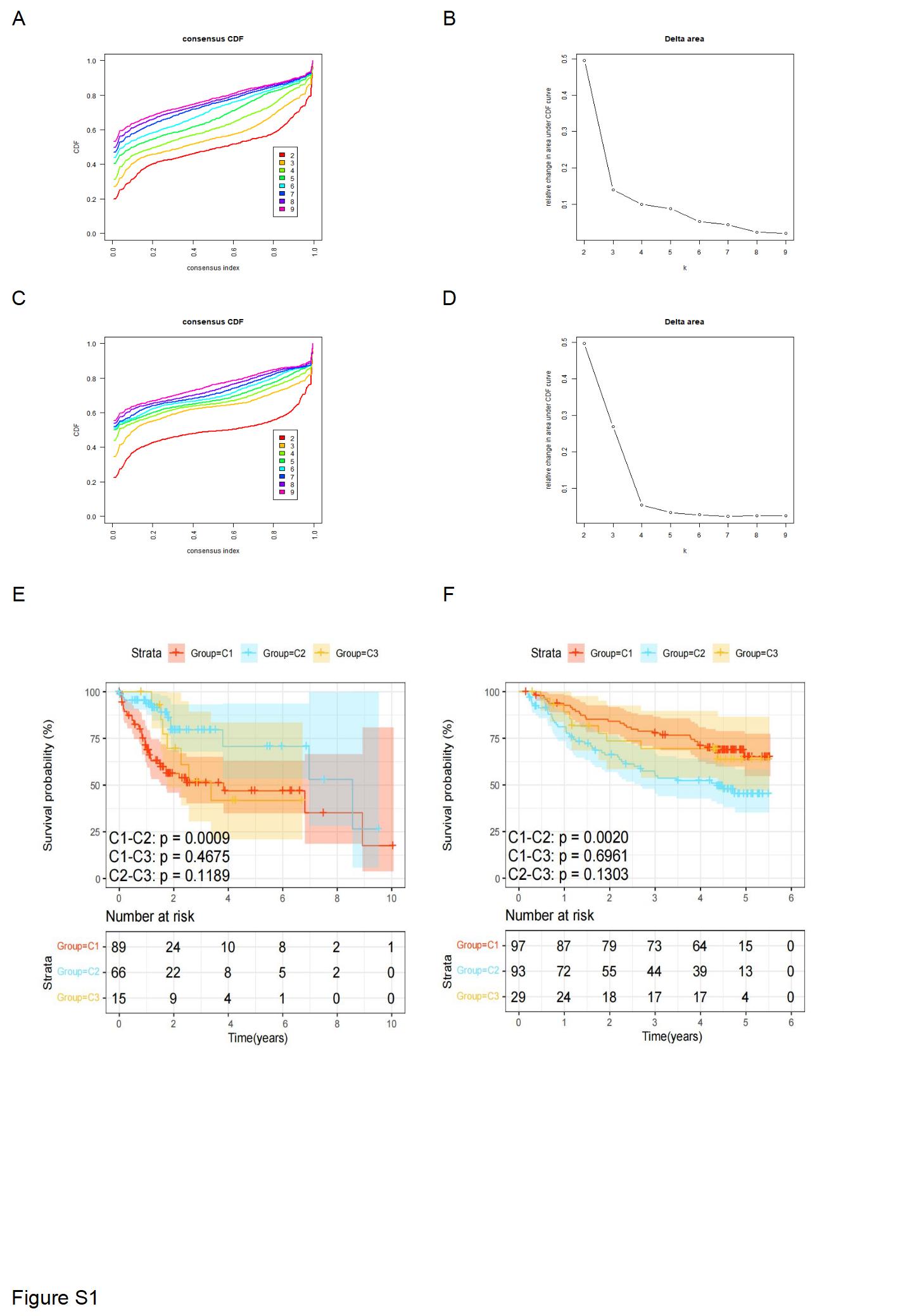


Figure S1. Unsupervised clustering. (A) The consistency clustering CDF curve for k = 2-9 in the TCGA training set. (B) The relative change of the area under the cumulative distribution function (CDF) for k =2-9 in the TCGA training set. (C) The consistency clustering CDF curve for k = 2-9 in the GSE14520. (D) The relative change of the area under the cumulative distribution function (CDF) for k =2-9 in the GSE14520. (E) KM curve analysis of overall survival at k = 3 in the TCGA training set. (F) KM curve analysis of overall survival at k = 3 in the GSE14520.


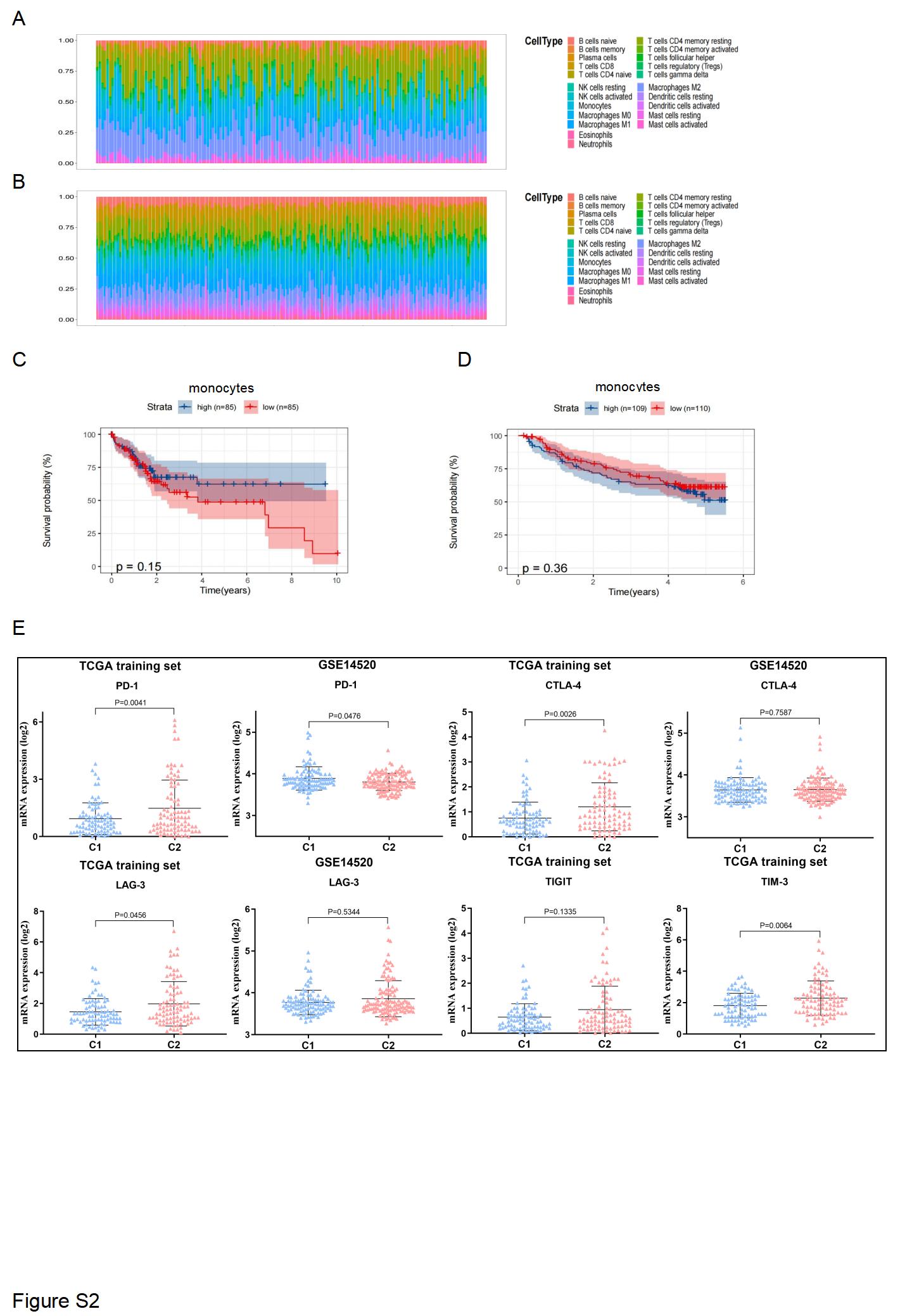


Figure S2. Analysis of infiltrated immune cells and checkpoints in TME. (A) The compositions of immune cells in the TCGA training set. (B) The compositions of immune cells in the GSE14520. (C) KM curve analysis of overall survival between high and low compositions of monocytes in the TCGA training set. (D) KM curve analysis of overall survival between high and low compositions of monocytes in the GSE14520. (E) Comparison of immune checkpoints between C1 and C2. *, P<0.05, **, P<0.01 and ***, P<0.001.


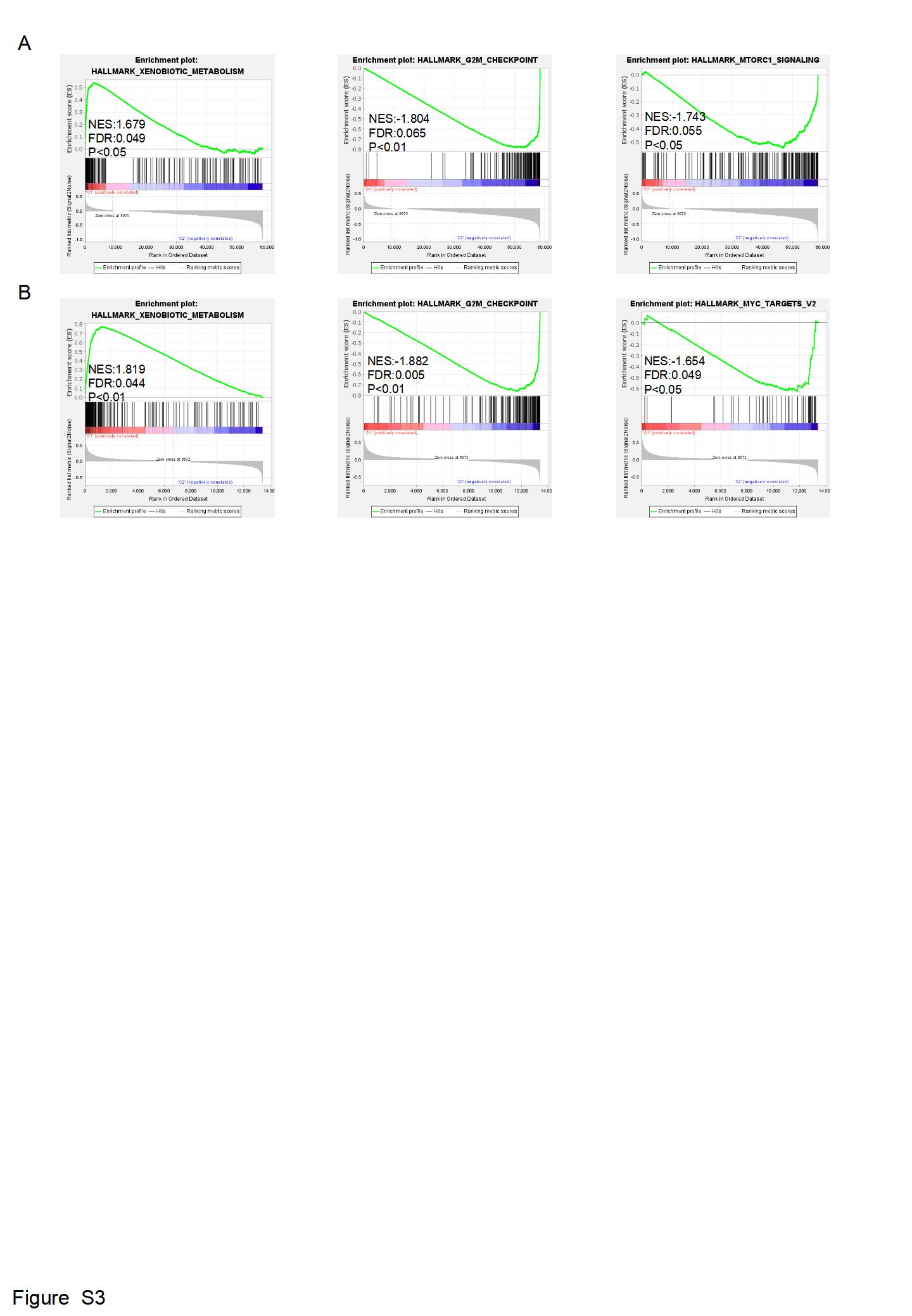


Figure S3. GSEA analysis. (A) GSEA analysis in TCGA training set. (B) GSEA analysis in GSE14520.


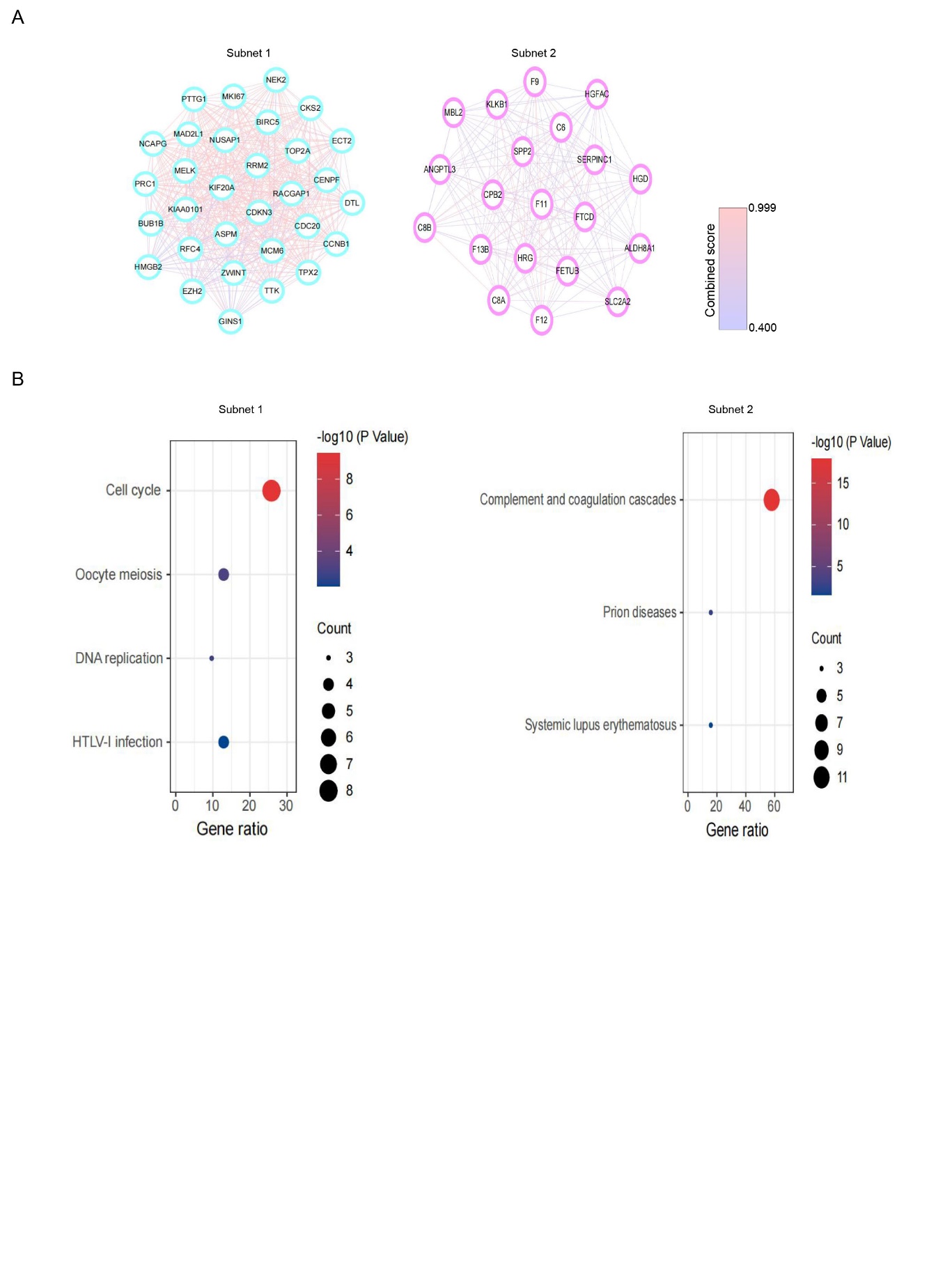


Figure S4. PPI network and KEGG analysis of subnet 1 and 2. (A) The PPI network of the subnet1 and 2. (B) KEGG analysis of the subnet1 and 2.


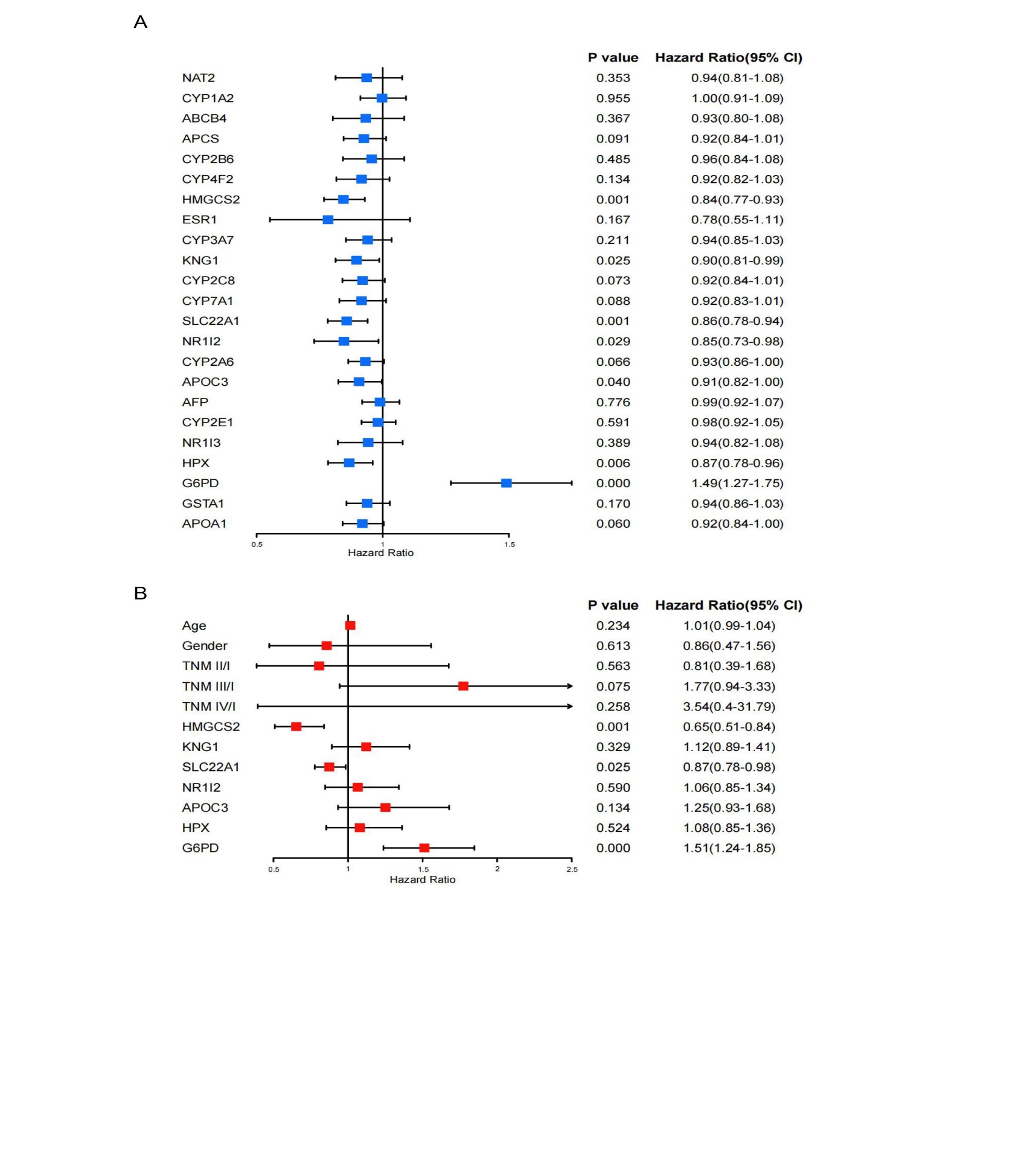


Figure S5. Univariate and multivariate Cox analysis. (A) Univariate Cox analysis of 23 hub genes in TCGA training set. (B) Multivariate Cox analysis in TCGA training set.


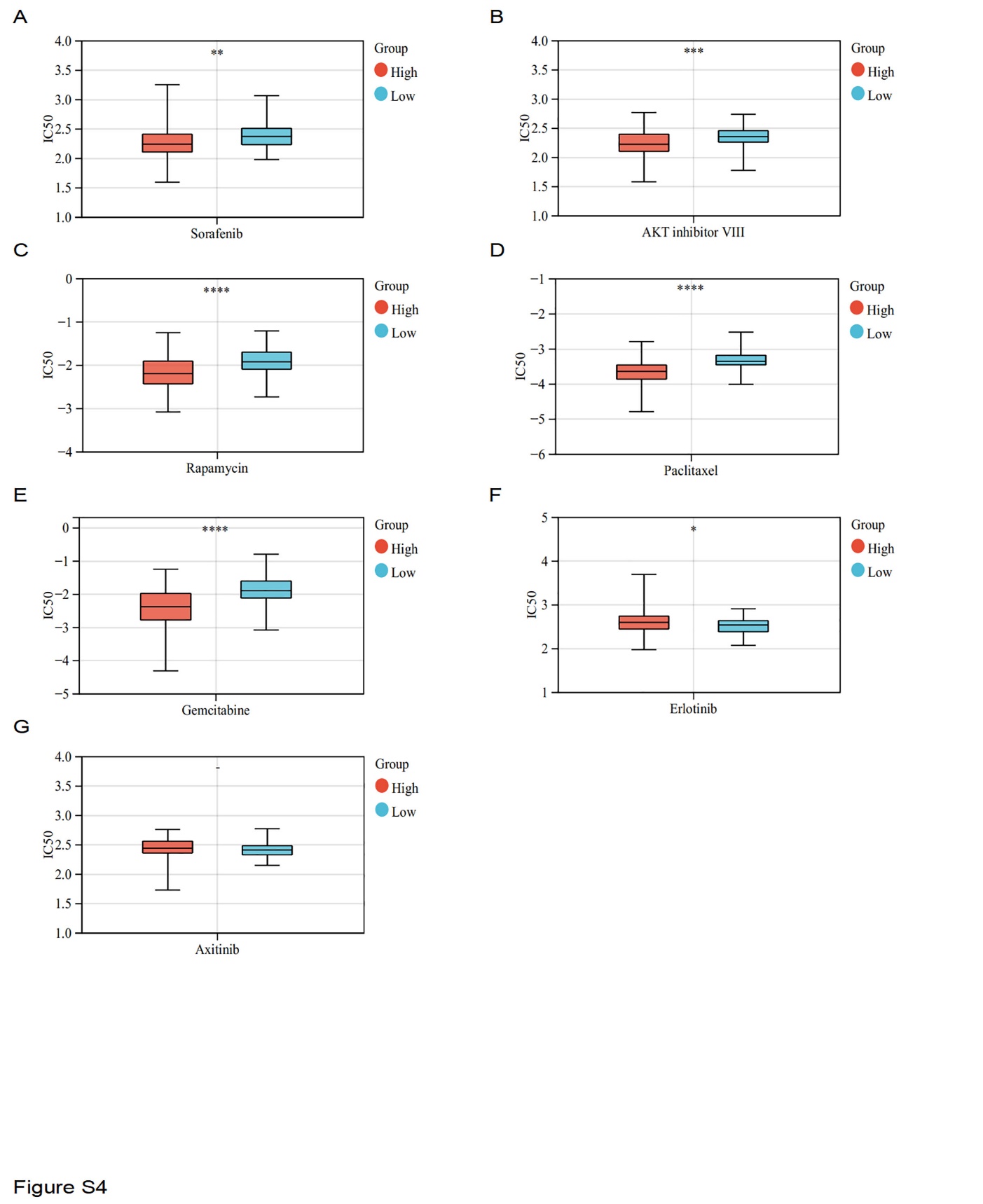


Figure S6. Chemosensitivity between high/low- risk groups in the TCGA training set. (A) Sorafenib. (B) AKT inhibitor VIII. (C) Rapamycin. (D) Paclitaxel. (E) Gemcitabine. (F) Erlotinib. (G) Axitinib.


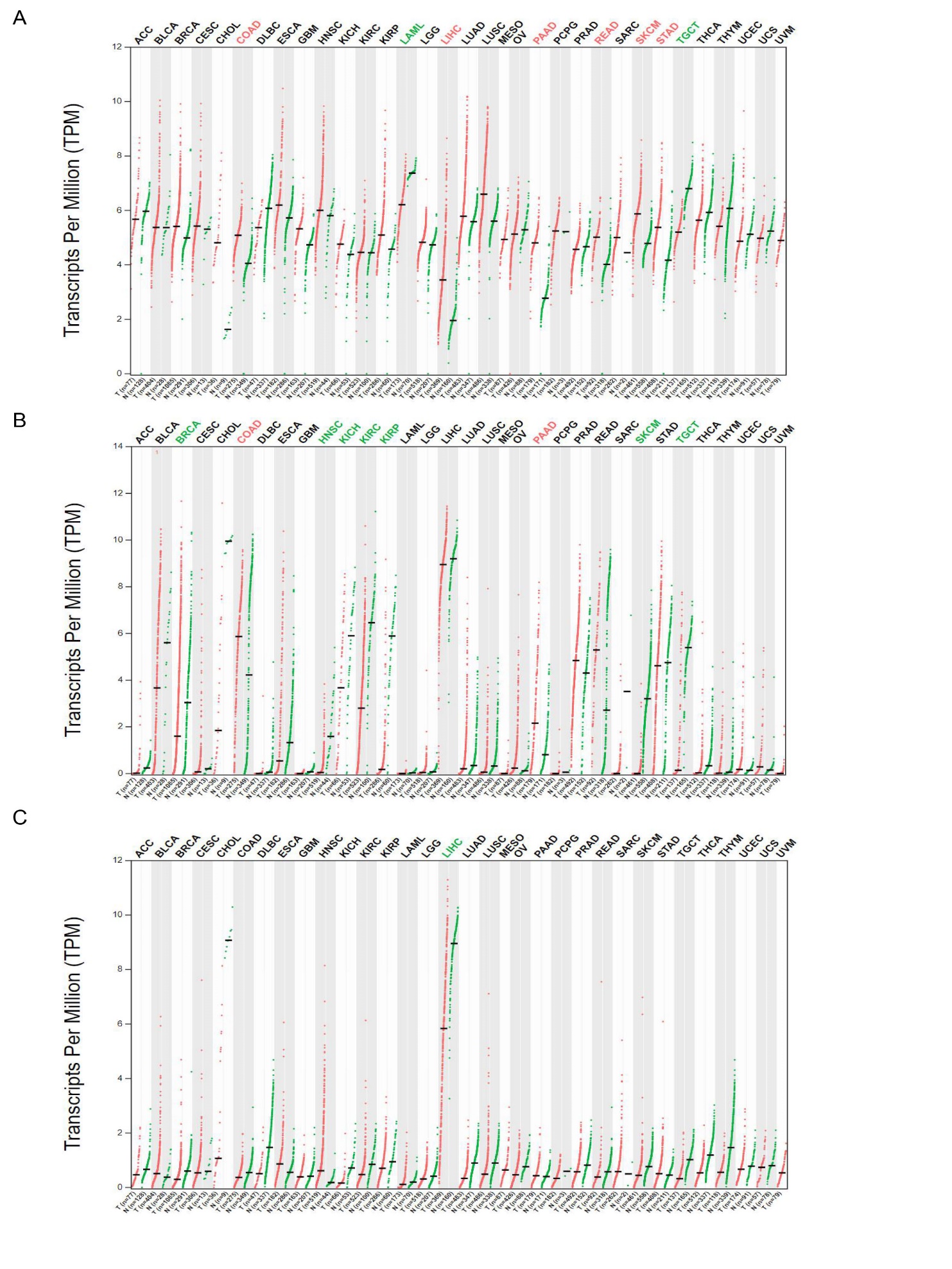


Figure S7. mRNA Expression of 3 genes in pan-cancers. (A) The mRNA expression levels of G6PD through GEPIA. (B) The mRNA expression levels of HMGCS2 through GEPIA. (C) The mRNA expression levels of SLC22A1 through GEPIA. Color represents statistical significance. Red represents high expression in cancer and green represents low expression in cancer.

|  | TCGA_trainning_set | | GSE14520 | |
| --- | --- | --- | --- | --- |
| Gene | HR(95%CI) | P value | HR(95%CI) | P value |
| IGF1 | 0.955(0.736-1.239) | 0.729 | 0.838(0.693-1.012) | 0.066 |
| MT1E | 0.998(0.903-1.103) | 0.971 | 0.944(0.849-1.049) | 0.283 |
| SQSTM1 | 1.43(1.139-1.794) | **0.002** | 1.023(0.881-1.187) | 0.769 |
| GHR | 0.754(0.619-0.919) | **0.005** | 0.87(0.776-0.975) | **0.017** |
| APOC3 | 0.913(0.832-1.002) | **0.04** | 0.852(0.764-0.973) | **0.018** |
| FOXM1 | 1.335(1.112-1.604) | **0.002** | 1.417(1.073-1.872) | **0.014** |
| GSTA4 | 1.105(0.895-1.364) | 0.351 | 1.153(0.937-1.418) | 0.179 |
| PON1 | 0.833(0.759-0.915) | **<0.001** | 0.817(0.706-0.945) | **0.006** |
| FOS | 0.993(0.851-1.159) | 0.933 | 1.042(0.901-1.206) | 0.578 |
| TOP2A | 1.264(1.071-1.493) | **0.006** | 1.275(1.086-1.497) | **0.003** |
| FEN1 | 1.372(1.078-1.747) | **0.01** | 1.431(1.098-1.865) | **0.008** |
| HELLS | 1.206(1.018-1.428) | **0.018** | 1.3(1.057-1.599) | **0.013** |
| CETP | 0.906(0.707-1.16) | 0.432 | 0.766(0.517-1.136) | 0.185 |
| EGR1 | 0.956(0.815-1.12) | 0.576 | 1(0.831-1.202) | 0.998 |
| BUB1B | 1.417(1.146-1.752) | **0.001** | 1.278(1.05-1.554) | **0.014** |
| MSRA | 0.785(0.59-1.044) | 0.096 | 0.697(0.566-0.86) | **0.001** |
| LEPR | 1.01(0.875-1.166) | 0.89 | 0.84(0.741-0.953) | **0.007** |
| PPARGC1A | 0.794(0.667-0.947) | **0.01** | 0.804(0.685-0.944) | **0.008** |
| SERPINE1 | 1.112(0.964-1.283 | 0.145 | 1.345(1.171-1.545) | **<0.001** |
| SOCS2 | 0.646(0.501-0.833) | **0.001** | 0.774(0.576-1.041) | 0.09 |
| CAT | 0.866(0.696-1.079) | 0.2 | 0.438(0.246-0.78) | **0.005** |
| PCNA | 1.482(1.137-1.933) | **0.004** | 1.141(0.869-1.498) | 0.343 |
| PDGFRA | 0.978(0.797-1.2) | 0.833 | 1.02(0.907-1.147) | 0.744 |
| PCK1 | 0.908(0.822-1.002) | 0.054 | 0.879(0.804-0.96) | **0.004** |
| PRKDC | 1.515(1.131-2.029) | **0.005** | 1.29(1.007-1.652) | **0.044** |
| ESR1 | 0.783(0.553-1.108) | 0.167 | 0.746(0.589-0.943) | **0.014** |
| IGFBP3 | 1.147(0.968-1.361) | 0.114 | 1.232(1.055-1.44) | **0.009** |
| LMNA | 1.52(1.096-2.11) | **0.012** | 1.215(0.847-1.744) | 0.29 |
| CDKN2A | 1.176(0.988-1.401) | 0.069 | 1.133(0.941-1.364) | 0.187 |
| H2AFX | 1.483(1.165-1.886) | **0.001** | 1.389(1.091-1.768) | **0.008** |
| CCNA2 | 1.392(1.151-1.684) | **0.001** | 1.116(0.904-1.377) | 0.307 |
| AR | 0.92(0.783-1.081) | 0.311 | 0.736(0.564-0.961) | **0.024** |
| HMGB2 | 1.446(1.151-1.817) | **0.002** | 1.215(0.985-1.5) | 0.069 |
| PARP1 | 1.322(0.966-1.809) | 0.081 | 1.205(0.817-1.776) | 0.347 |

**Table S1. Univariate Cox analysis.**

**Table S2. Tumor abbreviations note in TCGA.**

| TCGA | Detail | TCGA | Detail |
| --- | --- | --- | --- |
| ACC | Adrenocortical carcinoma | LUSC | Lung squamous cell carcinoma |
| BLCA | Bladder Urothelial Carcinoma | MESO | Mesothelioma |
| BRCA | Breast invasive carcinoma | OV | Ovarian serous cystadenocarcinoma |
| CESC | Cervical squamous cell carcinoma and endocervical adenocarcinoma | PAAD | Pancreatic adenocarcinoma |
| CHOL | Cholangio carcinoma | PCPG | Pheochromocytoma and Paraganglioma |
| COAD | Colon adenocarcinoma | PRAD | Prostate adenocarcinoma |
| DLBC | Lymphoid Neoplasm Diffuse Large B-cell Lymphoma | READ | Rectum adenocarcinoma |
| ESCA | Esophageal carcinoma | SARC | Sarcoma |
| GBM | Glioblastoma multiforme | SKCM | Skin Cutaneous Melanoma |
| HNSC | Head and Neck squamous cell carcinoma | STAD | Stomach adenocarcinoma |
| KICH | Kidney Chromophobe | TGCT | Testicular Germ Cell Tumors |
| KIRC | Kidney renal clear cell carcinoma | THCA | Thyroid carcinoma |
| KIRP | Kidney renal papillary cell carcinoma | THYM | Thymoma |
| LAML | Acute Myeloid Leukemia | UCEC | Uterine Corpus Endometrial Carcinoma |
| LGG | Brain Lower Grade Glioma | UCS | Uterine Carcinosarcoma |
| LIHC | Liver hepatocellular carcinoma | UVM | Uveal Melanoma |
| LUAD | Lung adenocarcinoma |  |  |
